# CRISPR/Cas9 based *mlo*-mediated resistance against *Podosphaera xanthii* in cucumber (*Cucumis sativus* L.)

**DOI:** 10.3389/fpls.2022.1081506

**Published:** 2022-12-19

**Authors:** Mumin Ibrahim Tek, Ozer Calis, Hakan Fidan, Mehraj D. Shah, Sefanur Celik, Shabir Hussain Wani

**Affiliations:** ^1^ Plant Protection Department, Faculty of Agriculture, Akdeniz University, Antalya, Türkiye; ^2^ Plant Virology and Molecular Pathology Laboratory, Division of Plant Pathology, Sher-e-Kashmir University of Agricultural Sciences and Technology of Kashmir, Srinagar, Jammu and Kashmir, India; ^3^ Mountain Research Centre for Field Crops, Khudwani, Sher-e-Kashmir University of Agricultural Sciences and Technology of Kashmir, Srinagar, Jammu and Kashmir, India

**Keywords:** cucumber, CsaMLO, defense response, plant susceptibility, powdery mildew, CRISPR/Cas9

## Abstract

Powdery mildews (PM) are common and severe pathogen groups that threaten plants, and PM resistance is complex and polygenic in cucumbers. Previously *mlo*-based resistance was reported in various plants, including cucumber, with generated loss-of *CsaMLO* function mutants. However, *mlo*-based resistance in cucumber is also complex and involves additional mechanisms such as hypersensitive response (HR) and papillae formation. For this reason, we focused on determining the *mlo*-based powdery mildew resistance mechanism in cucumber. CRISPR/Cas9 was used in the present study to generate loss-of-function mutants for *CsaMLO1*, *CsaMLO8*, and *CsaMLO11* of PM susceptible ADR27 cucumber inbred lines and *CsaMLO* mutants were obtained and validated. Trypan Blue and DAB staining were performed to detect *Podosphaera xanthii* germination/penetration rates and accumulation of Reactive Oxygen Species (ROS). Our results indicate that PM-susceptibility associated *CsaMLO*s in cucumber are negative regulators in different defense mechanisms against powdery mildew at early and late stages of infection. Further, the experiment results indicated that *CsaMLO8* mutation-based resistance was associated with the pre-invasive response, while *CsaMLO1* and *CsaMLO11* could be negative regulators in the post-invasive defense response in cucumber against *P*. *xanthii*. Although the loss-of *CsaMLO8* function confers the highest penetration resistance, *CsaMLO1* and *CsaMLO11* double mutations could be potential candidates for HR-based resistance against PM pathogen in cucumber. These results highlighted the crucial role of CRISPR/Cas9 to develop PM resistant cucumber cultivars, possessing strong pre-invasive defense with *CsaMLO8* or post-invasive with *CsaMLO1*/*CsaMLO11* mutations.

## Introduction

*Podosphaera xanthii* and *Golovinomyces cichoracearum* are the most common pathogens responsible for PM disease in cucumber (*Cucumis sativus* L.) Especially, *P. xanthii* is a serious pathogen in cucurbits, including *C. sativus* (Zitter et al., 1996). They are managed by fungicides in commercial agricultural production, but these chemicals affect human health and result in serious environmental problems (Xu et al., 2017). Also, PM pathogens could acquire resistance to fungicides with the excessive use of chemical fungicides which will be a more serious problem. Therefore, using PM-resistant cultivars is the most eco-friendly and durable management method for these pathogens. However, PM resistance is complex and polygenic and involves diverse plant defense mechanisms and signaling pathways (Nie et al., 2015). Generating PM-resistant cultivars with introgressed resistance (*R*) genes from wild form of cultivars is challenging for the breeders. Another concern is that the pathogen could overcome the resistance due to the mutations in avirulence genes even if the PM resistant cultivars are generated using long-term and tiresome backcrossing and inbreeding procedures.

Several susceptibility (*S*) genes were identified as an alternative for *R-*mediated resistance in the last decades, and it was supposed that the loss of *S*-gene function with controlled or naturally occurring mutations could confer durable and broad-spectrum resistance against various diseases in plants (de Almeida Engler et al., 2005). The proteins encoded by *S*-genes cause host recognition by the pathogen, defense suppression, and facilitate penetration (van Schie and Takken, 2014). These proteins are critical for disease caused by biotrophic pathogens such as PM and downy mildew because their vitality is directly associated with host interactions (Eckardt, 2002). *MLO*s are the most known *S*-genes responsible for PM-susceptibility in various plants. Barley *mlo* is one of the first identified genes associated with PM, and the loss of *mlo* function still confers the broad-spectrum and durable resistance against *Blumeria graminis* f.sp *hordei* (*Bgh*). Also, various research groups have reported *mlo*-mediated resistance in other plants such as *Arabidopsis thaliana* (Consonni et al., 2006), *Triticum aestivum* (Konishi et al., 2010), *Solanum lycopersicum* (Bai et al., 2008), *Pisum sativum* (Humphry et al., 2011), and *Cucumis sativus* (Berg et al., 2017) with phylogenetic analyses, and they revealed the fact that natural or controlled mutations on these homologs decrease PM susceptibility or confer higher resistance against PM (Jiwan et al., 2013; Zhou et al., 2013; Acevedo-Garcia et al., 2014).

*mlo*-based resistance contains different pathways and is associated with other defense mechanisms like *R-*gene-mediated PM resistance in various plants (Xu et al., 2019). For instance, papilla formation is a robust response against PM pathogens and is controlled by PENETRATION (PEN) and SNAREs VESICLE-ASSOCIATED MEM-BRANE PROTEINs (VAMPs) in *A. thaliana* (Assaad et al., 2004; Kwon et al., 2008; Böhlenius et al., 2010). The papilla formation is based on the accumulation of defense-related elements, such as cell-wall proteins, at the pathogen attack site (Schmelzer, 2002; Hückelhoven and Panstruga, 2011). This mechanism confers the resistance not only to PM but also to the avirulent/virulent fungi at the Microbe/Pathogen-Associated Molecular Pattern (M/PAMP) Triggered Immunity (PTI) stage, which is the host’s first layer defense response (De Wit et al., 2009). Membrane vesicles, reactive oxygen species (ROS), and phenolic compounds contribute to host cell wall reinforcement and prevent invasion (Kuhn et al., 2016). Callose is another significant compound for this pre-invasive response in plants (Ellinger and Voigt, 2014).

Hypersensitive reaction (HR) is another robust defense response against PM pathogens and occurs in Effector Triggered Immunity (ETI) as a known second layer defense response (Jones and Dangl, 2006). The intercellular branching of the PM pathogens is restricted by Programmed Cell Death (PCD) with recognition of the pathogen by host receptor proteins such as receptor-like kinase (RLK) and leucine rich repeat receptor like kinases (LRK). Phytohormones and ROS also contribute to the regulation of these defense responses to restrict pathogen growth (Micali et al., 2008; Takken and Goverse, 2012; Cao et al., 2014; Serrano et al., 2014).

*R*-gene-mediated PM resistance is multilayer like other pathogen resistance in the host. Different signaling pathways and proteins contribute to the regulation of defense response in plants (Kuhn et al., 2016). But how can we classify *mlo*-mediated resistance against PM pathogens? Recently, researchers classified the *mlo*-mediated resistance as a part of the non-host resistance (NHR) in *A. thaliana*. Because PEN proteins, the major compounds of NHR in *A. thaliana* against PM pathogens, are necessary for the *AtMLO2*-mediated resistance (Humphry et al., 2006; Appiano et al., 2015). Although *mlo*-mediated resistance is associated with NHR for this reason, the defense mechanism is still in ambiguity, particularly in cucumber.

*C. sativus* has thirteen *CsaMLO* genes, and three of these genes (*CsaMLO1, CsaMLO8, and CsaMLO11*) were classified in Clade V associated with PM-susceptibility in dicots. It has been reported that when *CsaMLO1* and *CsaMLO11* are minor genes for PM susceptibility, *CsaMLO8* could be a major gene associated with PM susceptibility in cucumber. Also, till date there is no report on the naturally occurring mutation on *CsaMLO1* or *CsaMLO11* (Nie et al., 2015; Berg et al., 2015). Although several research groups have focused on understanding *CsaMLO11/CsaML*O11 or *CsaMLO8* mediated resistance in cucumber, the defense mechanism is still unclear. Moreover, CRISPR/Cas9 for knock-out *CsaMLO1, CsaMLO8*, and *CsaMLO11* was suggested to determine *mlo-*mediated defense mechanism in cucumber (Berg et al., 2017).

We have previously determined the PM defense mechanism in cucumber using RT-PCR and found that callose-dependent cell-wall reinforcement was a more robust response than HR in our genotypes (Tek and Calis, 2022). Therefore, we presume that the major PM-susceptibility gene *CsaMLO8* could be responsible for regulating cell wall modifications negatively at pre-invasion stage. Also, we want to emphasize on the effects of the loss of function for Clade V genes in cucumber, *CsaMLO1*, and *CsaMLO11*, in defense response. Because the loss-of *CsaMLO* function is effect is still not clear in cucumber even if there are single *CsaMLO* mutant studies reported. Therefore, we used various types of loss-of *CsaMLO* function mutants such as triple and double mutants to determine *mlo*-mediated defense mechanism in cucumber. The study was carried out to reveal contribution of the *CsaMLO1, CsaMLO8*, and *CsaMLO11* mutations for pre or post invasive responses against *P*. *xanthii*. Loss of function cucumber mutants were developed to investigate defense mechanism such as penetration resistance at early stage of the infection or hypersensitive reaction in plants by using CRISPR/Cas9. Further, ROS (Reactive Oxygen Species) accumulation such as H_2_O_2_ (Hydrogen Peroxide) associated with plant defense system against different plant pathogens was investigated to classify defense response which linked Clade V *CsaMLO* mutations in cucumber.

## Material and methods

### gRNAs selection and vector transformation

**Figure 1:**
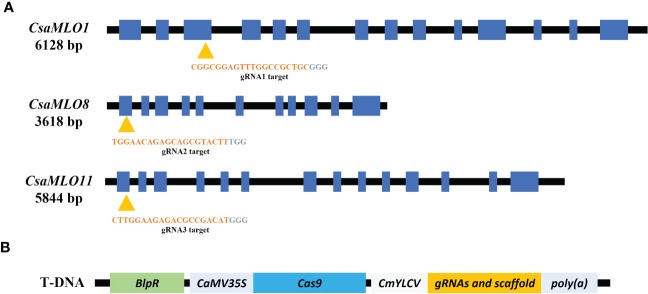
Specific gRNAs chosen from the coding site of Clade V *CsaMLO* (CDS) in cucumber and pDIRECT_23C plasmid. **(A)** gRNA1 for *CsaMLO1*, gRNA2 for *CsaMLO8*, and gRNA3 for *CsaMLO11* were selected by using CRISPR-GE. **(B)** Selected gRNAs were amplified with PCR and cloned into pDIRECT_23C’s. Cestrum yellow leaf curling virus promoter (CmYLCV) was used for gRNAs expression and bialaphos phosphinothricin resistance (BlpR) was used to transgenic plant selection. Also, pDIRECT_23C contains Cauliflower Mosaic Virus 35S promoter (CaMV35) for Cas9.

Three gRNAs have been selected for the *CsaMLO1, CsaMLO8*, and *CsaMLO11* by using CRISPR-GE (Xie et al., 2017) for Cas9 recognition in cucumber. The gRNAs were chosen from the third exon of *CsaMLO1* (Cucsa.207280) and the first exon of *CsaMLO8* (Cucsa.207280) and *CsaMLO11* (Cucsa.190600) sequences from Phytozome v12 (**Figure 1A**). pDIRECT_23:CmYLCV_gRNA1_gRNA2_gRNA3 plasmid (**Figure 1B**) was assembled from the described Golden Gate Assembly (Čermák et al., 2017). CmYLCV, CSY_gRNA, REP_gRNA, and csy_term oligonucleotides (**Supplementary Table 1**) were used in PCR to amplify gRNAs and gRNA scaffolds for each gRNAs. High Fidelity Phusion Taq (Thermo) polymerase was used in reaction mixture according to the manufacturer protocol. PCR was conducted in; pre-denaturation at 98°C for 30 secs, 30 cycle; denaturation at 98°C for 10 secs, annealing at 65 °C for 30 secs, elongation at 72 °C for 15 secs, and final elongation at 72°C for 5 mins conditions to amplify gRNA:gRNA_scaffold and pDIRECT_23C was used as a template. 50 ng pDIRECT_23C (**Supplementary Figure 1**), 0.5 µl of diluted (ten times) PCR products, 0.5 µl *SapI*, 0.5 µl *Esp3I*, 1 µl T7 DNA ligase, 10 µl 2x T7 DNA ligase buffer, and 7 µl ddH_2_O were used in Golden Gate reactions. The ligation was completed in 10 cycles at 37°C for 5 minutes and 25°C for 10 minutes. *Pme*I and *Pml*I restriction enzymes were used to confirm the plasmid and transferred to *E. coli* DH5α (**Supplementary Figure 2**). The transformation plasmid was isolated from DH5α after validation with kanamycin (50mg/L) and ccdB selection. Transformation plasmid was transferred to electrocompetent *Agrobacterium tumefaciens* EHA105 by electroporation. PC primers (annealing at 57°C) were used in colony PCR to confirm transformation of EHA105.

### Transformation and plant regeneration

Cotyledons of PM-susceptible cucumber line ADR27 were used as explants for *A. tumefaciens* EHA105-mediated transformation. The transformation and regeneration procedure were conducted as previously described (Zhang et al., 2017), with slight modifications. ADR27 inbred line’s seeds were kept in tap water for 2 hours and the seed coats were removed. Seed surfaces were sterilized using 75% ethanol for 30 seconds. Further, commercial bleach (3% sodium hypochlorite solution) was used also for sterilization for 15 minutes after the ethanol was drained out. After rinsing three times with deionized water and drying on sterile filter paper aseptically under laminar airflow cabinet, the seeds were placed in a petri dish containing plant germination medium (Murashige Skoog with 3% sucrose). Meanwhile, EHA-105 was incubated in Luria-Bertani (LB) broth containing 100 mg l^-1^ rifampicin and 50 mg l^-1^ kanamycin for 16-24 hours at 28°C and at 200 rpm. Bacterial cells were transferred to 50 ml of LB broth and incubated until the OD at 600 nm reached 0.6-0.8. After centrifugation at 4000 g for 10 minutes, the bacterial pellet was diluted in MS liquid to OD_600 =_ 0.25. Cotyledon explants were taken from a germination medium using sterile forceps and were prepared for transformation using a sterile scalpel. Prepared explants were transferred to the EHA105-containing liquid MS, incubated for 15 minutes for agroinfiltration, and placed in a co-cultivation Medium (MS with 3% sucrose, 2 mg ml^-1^ BAP, 2 mg ml^-1^ ABA, and 200 μM acetosyringone) at 28°C for two days in the dark keeping the abaxial surface in contact with the culture medium. The explants were then transferred onto a shooting medium (MS with 3% sucrose, 2 mg ml^-1^ BAP, 2 mg ml^-1^ ABA, 150 mg l^-1^ Timentin) containing 1 mg ml^-1^ glufosinate-ammonium to select transformant plants. After 2-3 weeks of incubation at 25°C under 16-hour photoperiod, the resulting shoots were transferred to glass jars containing a rooting medium (MS with 3% sucrose, 150 mg l^-1^ timentin and 1 mg ml^-1^ glufosinate-ammonium). After 2-4 weeks, the rooted plants were transferred to pots containing wet compost, covered with a zip bag, and grown for 6-8 weeks at 25°C/18°C and 16 hours photoperiod.

### Development of T1 plants

DNA was isolated from T0 plants using the GeneJET Genomic DNA Purification Kit, and transformation was confirmed by using Cas9 primers (**Supplementary Table 1**). After acclimatization, 0.1 M silver nitrate was sprayed on newly formed shoots to form male flowers for selfing. The transgenic T0 ADR27 male flowers, which opened on the same day, were crossed with female flowers. After the fruits ripened and matured, the T1 seeds were harvested, washed with 70% ethanol, dried, and stored at 4°C.

### Analysis of mutations in T1 plants

The T1 seeds were germinated in pots containing wet compost. Genomic DNA was extracted from the T1 cotyledons using GeneJET Genomic DNA Purification Kit as per manufacturer’s instructions (Thermo Fisher, Scientific, USA). Meanwhile, WT-specific forward primers were designed from downstream and upstream of the PAM sequence in for *CsaMLO1, CsaMLO8*, and *CsaMLO11*. Reverse primers were designed from GC-rich regions of these genes with high-annealing temperatures to increase specificity. The method is previously described as an “annealing at critical temperature PCR” (ACT-PCR) by Wang and Wang, 2019. Critical temperatures were determined with the gradient-PCR (60°C to 72°C) by using DNA isolated from WT-ADR27. PCR was carried out under the following conditions: initial denaturation at 94°C for 2 minutes, denaturation at 94°C for 30 seconds, annealing at 68°C (for *CsaMLO1* and *CsaMLO11*) and 65°C (for *CsaMLO8*) for 30 seconds, elongation at 72°C for 45 seconds; 30 cycle and final elongation at 72°C for 5 minutes to detect homozygous mutant. The PCR products were visualized after the agarose gel (1.5%) electrophoresis. The DNA from homozygous mutant plants that did not show amplification in the PCR using these primers could be attributed to the deletion or insertion mutation on the PAM sequence.

### Determination of the *CsaMLO* mutation types

**Figure 2:**
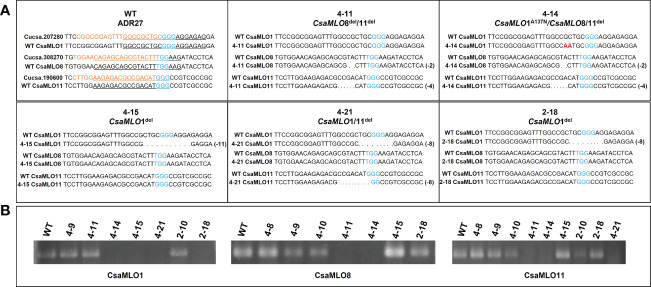
Nucleotide sequence alignment of *CsaMLO*s. Clade V *CsaMLOs* of WT-ADR27 were aligned with the reference sequence to detect any genotypic differentiation, and the underlined nucleotides on the alignment shows the PCR primers for detection of homozygous mutants; **(A)** the sequence results indicated that 4-11 has deletion mutations on *CsaMLO8* (2 bp) and *CsaMLO11* (4 bp) even if there is no mutation on the *CsaMLO*1. Although 4-14 is triple mutant (2 bp del at *CsaMLO*8 and 4 bp del at *CsaMLO11*), there is substitution mutation on *CsaMLO*1 with 2 nucleotides change as “AA” rather than “GC” on the Cas9 cleavage site. 4-15 and 2-18 were determined as *CsaMLO1* ^del^ with 11 bp and 8 bp deletions as a result of NHEJ. Also 8 bp deletions were detected on 4-21’s *CsaMLO1* and *CsaMLO11*; **(B)** The PCR products were observed to be non-mutant genotype in terms of *CsaMLO*. However, there was no amplification in PCR results of mutant, because of homozygous deletion or substitution on the primer binding sites.

*CsaMLO1, CsaMLO8*, and *CsaMLO11* gRNA targets of selected mutant plants accordingly ACT-PCR results were amplified with PCR by using the CsaMLO-Seq primers (**Supplementary Table 1**). PCR were carried out; pre-denutaration at 94°C for 5 mins; denaturation at 94°C for 1 min, annealing at 58°C for 45 secs, elongation at 72°C for 45 secs and final elongation at 72°C for 5 mins. The products were then run on 1.5% agarose gel using gel electrophoresis for confirmation. Finally, the amplicon was purified and sequenced using the Sanger method by Macrogen (Madrid, Spain). The T1 plants and WT ADR27 gRNA target sequences were evaluated with alignment of WT and mutant *CsaMLO* genes. Mutations were detected for each sequenced plant and are presented in **Figure 2**. The introns sequences were cleaned with reference CoDing Sequence (CDS) alignment. Cucsa.207280, Cucsa.308270, and Cucsa.190600 Phytozome reference sequences were used for *CsaMLO1*, *CsaMLO8*, and *CsaMLO11* respectively. CDSs were converted to amino acid sequence in GeniousPrime (2021.2.2v) and protein sequences of mutant were aligned with WT-ADR27. Open Reading Frame (ORF) shifts and stop codon formations were determined according to the alignment result.

### Inoculation and evaluation of reactions against PM

*P. xanthii* conidiospores were harvested from the PM infected plants with a brush and suspended in distilled water to give 2x10^5^ spore ml^-1^ and sprayed three times onto T1 mutant and susceptible WT ADR27 cucumber leaves. Ten days after the inoculation, leaf samples were taken from the PM inoculated WT and mutant plants, boiled in 96% ethanol to remove the chlorophyll, and then stained with trypan blue (250 µg ml^-1^). The development of conidiophores and conidiospores was examined under microscope (Leica DM500). Conidiospores were counted on five leaves for each plant within 100 spores at a 0.02 mm^2^ area for detection of spore germination and germination/penetration rates were compared between susceptible WT-ADR27 and mutants. Determination of ROS accumulation was carried out by staining leaves with DAB combined with Trypan Blue (Thordal-Christensen et al., 1997). For this, the PM-inoculated leaves were incubated for 6 hours in DAB (1%) solution and boiled in 96% ethanol. Leaves were evaluated and scored using a modified DR (Disease Reaction) scale where 0 indicates no fungal growth and 4 indicates dense fungal growth (Adam and Somerville, 1996).

## Results

### Plant regeneration and development of T1 mutants

After germination of ADR27 seeds, cotyledons were removed and used as explants for transformation using *A. tumefaciens* EHA 105 and were then transferred to co-cultivation medium. The explants were shifted to an MS medium containing 1 mg ml^-1^ glufosinate-ammonium for selection of transformants. The integration of T-DNA into plant genome was confirmed by PCR using Cas9 specific primers and 46 transgenic plants were regenerated at T0. Regeneration steps and T0 plants were given in **Supplementary Figure 3**. 96 T1 plants were also examined using ACT-PCR, and the result revealed that the mutation rates of the *CsaMLO* genes observed in the T1 generation were 25%, 2.08% and 19.79% for *CsaMLO1*, *CsaMLO8*, and *CsaMLO11*, respectively (**Supplementary Table 2**).

### In/dels in mutants

*CsaMLO1* did not amplify in PCR that was carried out with isolated DNA samples from 4-14, 4-15, 4-21, and 2-18 lines. *CsaMLO8* was amplified when the T1 plants, excluding 4-11 and 4-14 were used in PCR. Also, *CsaMLO11* did not amplify in 4-11, 4-14, and 4-21. The sequences of the gRNA’s target of these lines revealed that these lines have deletion and substitution mutations on the Cas9 cleavage site. 4-14 have 2 nucleotide substitution mutations on the upstream of the PAM motif. The deletion mutations were detected in the 4-15, 4-21, and 2-18 cucumber lines. The *CsaMLO1* deletion mutations in mutant lines were relatively larger than the *CsaMLO8* deletions. The small deletions (2 bp) are seen upstream of the PAM in the *CsaMLO8* of 4-11 and 4-14 T1 plants. Deletions were also detected in the *CsaMLO11* of 4-11, 4-14, and 4-21 T1 plants. The *CsaMLO11* deletions are 4 and 8 bp, and the location of the deletions differed from the PAM motif of *CsaMLO1*. The position of deletions ranged from downstream of the PAM to upstream in *CsaMLO1*, but the deletion was detected only upstream of the PAM in *CsaMLO11* (**Figure 2**).

### Early stop codon formation and amino acid substitution

**Figure 3:**
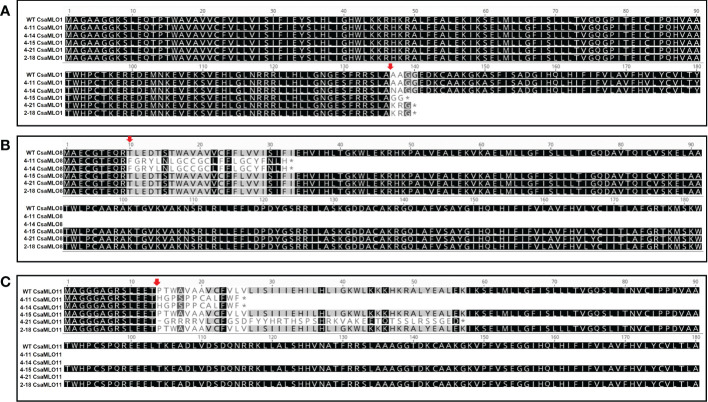
Amino acid alignment using sequences of mutants and WT plants. **(A)** When the deletions on the Cas9 cleavage (red arrow) cause stop codon formation on the 4-15’s *CsaMLO1* 139th amino acid; the stop codon formation was detected on 2-18 and 4-21 *CsaMLO1’s* 140th amino acid (3rd exon). The Alanine **(A)** was altered with Asparagine (N) at the 137^th^ amino acid as a result of the NHEJ caused by Cas9 cleavage at the gRNA1 target. **(B)** 2 bp deletions cause the unexpected stop codon formation on the 33^rd^ amino acid of *CsaMLO8* in 4-11 and 4-14. The target site of gRNA2 was on the first exon for *CsaMLO8* and the changed amino acid structures are at the Cas9 cleavage (10^th^ aa) site for this gene. The early stop codon formation caused the loss-of *CsaMLO8* function in 4-11 and 4-14. **(C)** The mutations seem to be responsible for the stop codon formation in *CsaMLO*11 and stop codon’s positions of 4-11 and 4-14’s *CsaMLO11* closer to that gRNA3 than 4-15 and 2-18 *CsaMLO11*. However, ORF shifts are initiated at gRNA3 position in whole *CsaMLO11* ^del^.

Most of the mutations occurred in different mutant plants caused early stop codon formation at the amino acid sequence except that in 4-14 mutant plant, two nucleotide mutations (GC to AA) in *CsaMLO*1 lead to alanine to asparagine change at the 137th amino acid as a result NHEJ (non-homologous end joining) after the Cas9 cleavage. The alanine was changed asparagine at the 137^th^ amino acid of *CsaMLO1* as a result NHEJ (non-homologous end joining) after the Cas9 cleavage. The stop codon formation was detected at the 140^th^ amino acid of 4-21 and 2-18’s *CsaMLO1* caused by 8 bp deletion, while the stop codon was also detected on 139^th^ amino acid of 4-15’s *CsaMLO1* caused by 11 bp deletion. The ORF shifts were detected on the *CsaMLO8* mutants 4-11 and 4-14 at Cas9 cleavage site and stop codon formation was detected at the 33^rd^ amino acid. ORF shift and stop codons also detected in *CsaMLO11* as a result of NHEJ. The stop codons were at the 26^th^ amino acid of 4-11 and 4-14 *CsaMLO11*, while stop codon formation appeared at 57^th^ amino acid of 4-21’s *CsaMLO11*. 4-11 (*CsaMLO8/11* ^del^), 4-14 (*CsaMLO1* ^A137N^/*CsaMLO*8/11^del^), 4-15 (*CsaMLO1* ^del^), 4-21 (*CsaMLO1*/11^del^), and 2-18 (*CsaMLO1* ^del^) were determined as loss of function mutant (**Figure 3**).

### Evaluation of mutants for *P. xanthii* resistance

**Figure 4:**
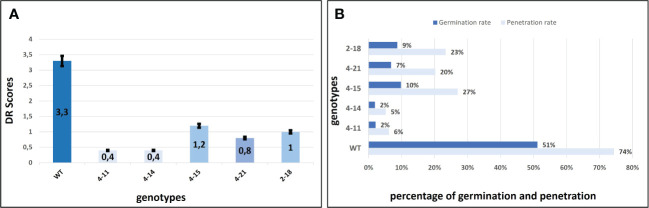
Evaluation of the DR scores against PM and *P. xanthi* penetration/germination rates at ten-day post-inoculation. **(A)** DR scores of wild-type ADR27 and mutant plants were generated means of the inoculated five leaves for each genotypes at 10 dpi (**Table 1**) and same colors indicated that there is no difference for DR scores statistically according to standard deviation bar. The lowest DR scores were observed in 4-11 (*CsaMLO8*/11^del^) and 4-14 (*CsaMLO1* ^A137N^/8/11^del^). The DR scores were statistically similar for 4-15 and 2-18 plants having *CsaMLO1* mutation. 4-21 was determined as *CsaMLO1*/11 ^del^ mutant and the DR score was lower than *CsaMLO1* ^del^ even if higher DR score than 4-11 and 4-14. **(B)** The strong penetration resistance is defined in 4-11 and 4-14 plants having loss of *CsaMLO8* function. Also, *P. xanthii* germination rate was lowest in these mutants. 4-15 and 2-18 (*CsaMLO1* ^del^) penetration and germination rates were similar with the *CsaMLO1*/11^del^. Percentages of the germination and penetration rates were created according to average of germinated and penetrated spores’ numbers from each genotype’s five leaves.

The *P. xanthii* was inoculated on 4-11 (*CsaMLO8*/11^del^), 4-14 (*CsaMLO1* ^A137N^/*CsaMLO*8/11^del^), 4-15 (*CsaMLO1* ^del^), 4-21 (*CsaMLO1*/11^del^), 2-18 (*CsaMLO1* ^del^), and WT cucumber plants after the determination of the mutation and mutation types. After ten days of inoculation, inoculated five leaves were examined closely for inoculated WT and mutant plants to determine DR scores (**Table 1**). Further, leaves were also harvested from the WT and mutant plants, chlorophyll was removed, and staining was done with Trypan Blue. Typical PM symptoms were apparent on WT ADR27 leaves. 4-15 and 2-18 mutants showed fewer symptoms on the inoculated leaves. The PM symptoms on 4-21 and 4-11 plants were less severe than in *CsaMLO1* ^del^ plants. Furthermore, there were no symptoms on the 4-14 plant. The microscopic observation results confirmed that the conidiospores had germinated massively and developed many new spores on the WT ADR27 plant. However, no new conidiophores or conidiospores were observed on the inoculated *CsaMLO8*/11^del^ and *CsaMLO1* ^A137N^/*CsamMLO8*/11^del^ (**Figure 4**). Low spore germination and limited mycelial growth was observed on the 4-11 and 4-14 plants.

**Table 1:** DR scores at 10 dpi on different PM-inoculated leaves.

Leaf	WT	4-11	4-14	4-15	4-21	2-18
1	3.50	0.50	0.50	1.50	1	1
2	3.50	0.50	0.50	1.00	0.50	1
3	3.50	0.50	0.00	1.50	0.50	1
4	30	0.50	0.50	1.00	1	1
5	30	0.00	0.50	1.00	1	1
Average	3.30	0.4	0.40	1.20	0.80	1

### ROS accumulation on *CsaMLO8*/11^del^ and *CsaMLO1* ^A137N^/CsaMLO8/11^del^

**Figure 5:**
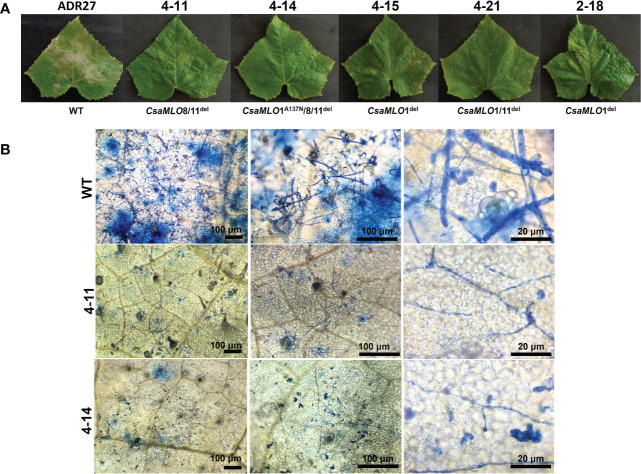
Powdery mildew symptoms and visualization of *P. xanthii* spores on WT/mutant leaves using Trypan Blue. **(A)** Powdery mildew symptoms on leaf surfaces of WT-ADR27 and *CsaMLO* mutants at 10 dpi. Typical symptoms could be seen on the WT leaves as a result massive propagation caused by *P. xanthii* after ten-days post inoculation. Restricted symptoms were detected on *P. xanthii* inoculated leaves of 4-15, 4-21, and 2-18 plants. Moreover, the hypersensitive response associated lesions were apparent on the leaves of *CsaMLO1* ^del^ and *CsaMLO1*/11^del^ plants. However, no symptoms appeared on the leaves of 4-11 and 4-14 plants after ten days of inoculation. **(B)** The epidermal surface of WT-ADR27 was covered with *P. xanthii* conidiophore and conidiospores that was newly formed from inoculated spores. Also, massive cell deaths were observed on the leaf mesophyll as a result of pathogen activity apparent from the blue color. However only mycelial growth was detected on the leaves of 4-11 and 4-14 plants, even if there was restricted germination rate.

**Figure 6:**
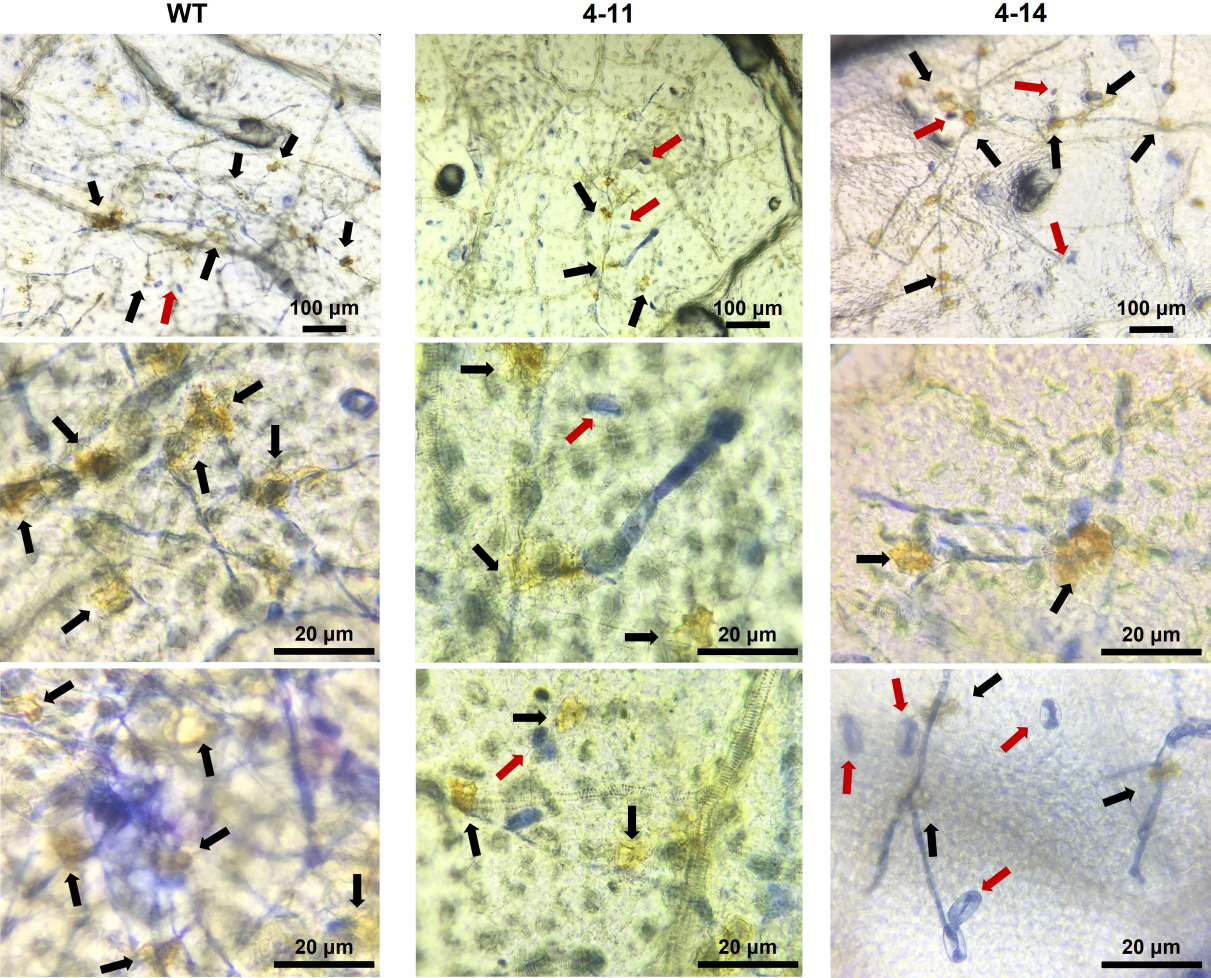
Combined DAB and Trypan blue staining to determine ROS accumulation on leaves of inoculated WT and mutant plants at 10 dpi. Although susceptible WT-ADR27 had highest *P. xanthi* germination rate, ROS accumulation (black arrow) was found more frequently on the leaves of these plant as compared 4-11 and 4-14. ROS accumulation was also found in 4-11 and 4-14 which are *CsaMLO8*/11^del^ and *CsaMLO1* ^A137N^/8/11^del^ mutants. However, ROS were accumulated in the *P. xanthii* mycelial growth site was detected in 4-11 and 4-14. Also, non-germinated spores (red arrow) were detected on mutant leaves on the contrary to WT.

Trypan blue staining revealed massive mesophyll cell death on ADR27 associated with PM infection. However, there were restricted death cells in 4-11 and 4-14 plants (**Figure 5**). The DAB and Trypan Blue combined staining also shows ROS accumulation on ADR27 and mutant plants. ROS accumulation was observed near the *P. xanthii* mycelium growth area of the leaves in both mutant and WT plants. Notably, on 4-14 mesophyll cells, the pathogen conidiospores that were unable to germinate were quite visible, even though there was no ROS accumulation and cell death. Combined staining also revealed that the mycelial growth was on wild-type ADR27 leaves, and ROS was accumulated in this growth area. The maximum ROS accumulation and *P. xanthii* development were on the WT ADR27 when compared with 4-11 and 4-14 (**Figure 6**).

## Discussion

*Podosphaera xanthii* is one of the important pathogens responsible for powdery mildew, which is thought to be affecting more than ten thousand plant species worldwide. The PM caused by *P. xanthii* is the most common and severe disease in cucurbits, including *C. sativus* (Zitter et al., 1996). Understanding the PM defense mechanism is the first and critical step to develop PM resistant cultivars. However, the generation of resistant cultivars is challenging because PM resistance is controlled by several genes (polygenic in nature) and involves complex defense pathways (Chen et al., 2009).

Cell wall reinforcement and HR are strong responses against PM pathogens. Significantly callose-associated cell wall reinforcement is one of the robust basal defense mechanisms found in plants against incompatible plant-microbe interaction (Ahmed et al., 2016). M/PAMPs are recognized by host receptors such as PRR and trigger the PTI, the first line of defense in plants, a defense mechanism activated to restrict the infection. However, PTI pressures the pathogen for mutation on effectors, and only successful pathogens could overcome the first obstacle (Thordal-Christensen, 2003). For this reason, we need to find new resistance sources to generate new resistant cultivars. The new sources (*R*-genes) could confer resistance against successful pathogens (overcame the first line) that trigger the ETI, and a hypersensitive reaction generally occurs at this stage (Jones and Dangl, 2006). However, *R*-gene-mediated resistance is broken, and new pathogen strains could overcome the resistance in a short time with a mutation on avirulence genes. Therefore, NHR is strongly suggested against plant pathogens to solve this infinity loop because of it is durable and broad-spectrum resistance.

It has been suggested that *S-*gene mutation mediated resistance as a novel approach for developing resistant cultivars to control plant disease. One of the most recognized and first discovered *S-*gene is the barley *mlo*. Naturally occurring mutations on this gene confer broad-spectrum and durable resistance against many *Bgh* isolates (Jørgensen, 1992). Unfortunately, *mlo*-mediated resistance is as complex as *R-*mediated PM resistance in various plants, till date very little is known about the *mlo*-mediated defense mechanism in various plants (Acevedo-Garcia et al., 2014). However, it has been shown that a calmodulin-binding domain of barley *mlo* increases susceptibility to PM, particularly in barley. Furthermore, the *MLO* have seven transmembrane domains, and one of these (the calmodulin-binding domain) is responsible for calmodulin-binding. The function of this protein is to block the Ca^+2^ and calmodulin interaction. The Ca^+2^ signal plays an essential role in recognizing the host by the PM pathogen at the early stage of the infection (Kim et al., 2002).

Phylogenetic analyses and transcriptomic studies have been focused on revealing *MLO* gene families in cucumber. Differentially Expressed Genes (DEGs) were determined in the loss-of-function mutants in cucumber. However, *mlo*-mediated resistance is still poorly understood in cucumber because of different defense responses that could occur against PM pathogens in cucumber, contrary to other plant species (Berg et al., 2015; Nie et al., 2015). Contrary to the *CsaMLO1* and *CsaMLO11* mutants in cucumber, mutant cucumber for *CsaMLO*8 was found in nature. Further, using the latest gene-editing techniques was suggested to determine *mlo*-mediated resistance in cucumber by obtaining different loss-of-*CsaMLO* function mutants such as triple or double mutants (Berg et al., 2017). Therefore, CRISPR/Cas9 was used to generate triple (*CsaMLO1* ^A137N^/*CsaMLO*8/11^del^), double (*CsaMLO1*/11^del^ and *CsaMLO8*/11^del^), and single mutant (*CsaMLO1* ^del^) cucumber lines and these lines were used in this study to determine *mlo*-based defense mechanisms in cucumber.

The T0 lines obtained after transformation were further tested with Cas9 primers to control T-DNA integration and 46 plants determined as transgenic. We screened the mutants which are obtained from transgenic T0 with a PCR-based approach (ACT-PCR) to determine homozygous mutants in T1 generations. The mutation rates in 96 T1 plants were 25%, 2.08% and 19.79% for *CsaMLO1*, *CsaMLO8*, and *CsaMLO11* respectively. The PCR-based approach could be the reason for low mutation rates in T1 reactions because the method allows the detection of only homozygous mutant plants.

The deletions mutations were detected on *the CsaMLO1* target sequence of 4-15 (11 del), 4-21 (8 del), and 2-18 (8 del), and detected deletions cause loss-of *CsaMLO1* function due to the formation of the unexpected stop codon formation at 139^th^ amino acid of 4-15’s *CsaMLO1*, and 140^th^ amino acid of 4-21 and 2-18’s *CsaMLO1*. Interestingly, two nucleotide substitution mutations at the Cas9 cleavage site of 4-14’s *CsaMLO1* caused amino acid substitution. The result is similar to amino acid substitution on barley, causing the previously reported loss-of-function mutation for barley’s *mlo* (Reinstädler et al., 2010). However, there was no difference between DR scores of 4-11 and 4-14 plants post PM inoculation. Two deletions were detected on *CsaMLO8* of 4-11 and 4-14, hence, these deletions resulted in the stop codon formation at 33^rd^ amino acid. Also, 4 and 8 bp deletions were seen on *CsaMLO11* of 4-11, 4-14, and 4-21 lines. To sum up, we report for the first-time triple mutants 4-14 (*CsaMLO1* ^A137N^/*CsaMLO*8/11^del^), double mutants 4-11 (*CsaMLO8*/11^del^), and 4-21 (*CsaMLO1*/11^del^) in cucumber lines using CRISPR/Cas9.

Although various research groups have generated *CsaMLO8* mutant cucumber lines using different methods and DEGs were determined with transcriptomic approaches, but the underlying mechanism of *mlo*-mediated resistance in cucumber is still unclear. Therefore, we performed this study to reveal the defense mechanism involved in the *mlo*-mediated resistance in cucumber. In this study, we focused on the *mlo*-mediated resistance mechanism, and PM reactions of 4-11 (*CsaMLO8*/11^del^), 4-14 (*CsaMLO1* ^A137N^/*CsaMLO*8/11^del^), 4-15 (*CsaMLO1* ^del^), 4-21 (*CsaMLO1*/11^del^), and 2-18 (*CsaMLO1* ^del^) were evaluated post-inoculation. The results from the symptoms caused by PM on mutant plants indicated that the loss of *CsaMLO8* function confers resistance against the PM. The results from the symptoms caused by PM on mutant plants indicated that the loss of *CsaMLO8* function confers resistance against the PM. DR scores were lowest in 4-11 and 4-14, with 0.4 DR scores among mutant plants, there is no statistical difference in terms of DR scores between 4-15 and 2-18, which are single *CsaMLO1* ^del^, and their DR scores were higher than *CsaMLO1/11* ^del^ 4-21’s DR score (0.8). The reason of the identical DR scores between 4-11 and 4-14 could associated with the early *CsaMLO8* mutation-mediated response against PM or *CsaMLO1* substitution mutation in 4-11 did not affect the resistance level in *CsaMLO8* ^del^ plants. Although, this result is not astonishing, the evaluation of the PM reactions shed light on the *mlo*-mediated defense mechanism in cucumber using the DAB and Trypan Blue staining.

Hypersensitive reaction-associated lesions were seen on the leaf surface 4-15 and 2-18 plants at ten days post-inoculation, and these lines also exhibited the least deletion mutations on *CsaMLO1*. Deletion mutations were also observed on *CsaMLO1* and *CsaMLO11* in 4-21. The PM symptoms were restricted with the HR in *CsaMLO1* ^del^ and *CsaMLO1*/11^del^. Although *P. xanthii* germination rate was higher than 4-11 and 4-14, our results have revealed that *CsaMLO1* and *CsaMLO11* could be negative regulators in post-invasive defense response against PM pathogen in cucumber. 4-11 and 4-14 plants recorded the lowest DR score after the PM inoculation, as expected, because of the *CsaMLO8* function, a major susceptibility gene for PM. Interestingly, there was no HR observed on the adaxial leaf surface of 4-11 and 4-14 plants, and there appeared no symptoms even after ten days of inoculation. From the microscopic observation it was quite visible that attached ungerminated *P. xanthii* spores on epidermal cells were staining blue after Trypan blue assay. Only a few spores germinated, and only mycelial growth was detected under the cucumber leaf cells. To our surprise, accumulation of ROS was detected during DAB staining, even in the absence of HR. However, ROS such as H_2_O_2_ are not only associated with HR but have been reported to be linked with callose-dependent cell-wall reinforcement, known as papilla formation (Kuźniak and Urbaneki, 2000). The localized ROS accumulation was also detected on inoculated and non-germinated *P. xanthii* conidiospores. The DAB combined Trypan blue staining result indicated that accumulated ROS could be associated with PM-triggered cell-wall reinforcement in 4-11 and 4-14 plants, but the accumulated ROS did not reduce the PM infection rate in WT. The lower *P. xanthii* germination and restricted mycelial growth indicate that the *CsaMLO8* could be a negative regulator in pre-invasive defense response. Previously, *CsaMLO*8 and callose interaction has been demonstrated. However, the loss-of-*CsaMLO8* function provides strong penetration resistance in cucumbers at early stage of infection even if there are loss-of-function mutations on *CsaMLO1* or *CsaMLO11*.

Finally, we can develop novel resistant cultivars by using state-of-the-art gene editing techniques without understanding the underlying mechanism of this type of resistance. However, we need to figure out the defense mechanism sourced by *S-*gene mutations in the plant to develop broad-spectrum and durable resistance. It is not possible that the pathogen can overcome the resistance provided by *S-*gene mutation because this resistance is similar to NHR, especially *mlo*-mediated resistance. However, it is difficult to claim that *S-*mediated resistance will not break in the near future. Therefore, investigating the defense mechanism with novel and diverse approaches could be effective for protecting agricultural production against yield and quality losses caused by plant pathogens.

## Conclusion

Three types of mutants i.e., triple mutant 4-14 (*CsaMLO1* ^A137N^/CsaMLO8/11^del^), double mutants 4-11 (*CsaM*LO8/*11* ^del^) and 4-21 (*CsaMLO1*/*CsaMLO11* ^del^), single mutants 4-15 and 2-18 (*CsaMLO1* ^del^) were generated in this study using the CRISPR/Cas9. Loss-of Clade V functions with diverse combinations were investigated to determine *mlo*-mediated resistance in cucumber. The results revealed that *CsaMLO1*/*CsaMLO11* mutations confer resistance with HR for post-invasive defense response. Loss of *CsaMLO8* function conferred higher resistance than other mutants as described previously, but we report here for the first time, that *CsaMLO8* is strongly associated with pre-invasive defense response even if there are other Clade V mutants such as in triple mutant. Hence, CRISPR/Cas9 could be used for the generation of PM-resistant cucumber cultivars, which have a strong pre-invasive defense with *CsaMLO8* or post-invasive with *CsaMLO1*/*CsaMLO11* mutations.

## Data availability statement

Sequence data of the cucumber CsaMLO1 (Cusca.207280), CsaMLO8 (Cusca.308270), and CsaMLO11 (Cusca.190600) from Phytozome (https://phytozome.jgi.doe.gov/) has been used for selection of the gRNAs. CsaMLO sequences of the mutant and wild type were uploaded to NCBI GenBank (ON528937-ON528951); ON528941 for WT-CsaMLO1, ON528942 for 4-14’s CsaMLO1, ON528943 for 4-15’s CsaMLO1, ON528944 for 4-21’s CsaMLO1, ON528946 for 2-12’s CsaMLO1, ON528937 for WT-CsaMLO8, ON528938 for 4-11’s CsaMLO8, ON528940 for 4-14’s CsaMLO8, ON528948 for WT-CsaMLO11, ON528949 for 4-11’s CsaMLO11, ON528950 for 4-14’s CsaMLO11, and ON528951 for 4-21’s CsaMLO11.

## Author contributions

Conceptualization: OC and SW. Data curation: MT, MS, and SC. Formal analysis: MT, MS, and SC. Funding acquisition: OC. Methodology: MT. Project administration: OC and HF. Resources: SW. Supervision: OC, HF. Writing – original draft: MT. Writing – review & editing: HF, SW. All authors contributed to the article and approved the submitted version.
