# Supplementary material for: CRISPR/Cas9 based mlo-mediated resistance against Podosphaera xanthii in cucumber (Cucumis sativus L.)
Source: Front Plant Sci. 2022 Dec 19;13:1081506. doi: 10.3389/fpls.2022.1081506 (PMC9806270; doi:10.3389/fpls.2022.1081506)
Supplement: Supplementary file 1 [file DataSheet_1.zip › Supplementary file26112022.docx]

Supplementary Material


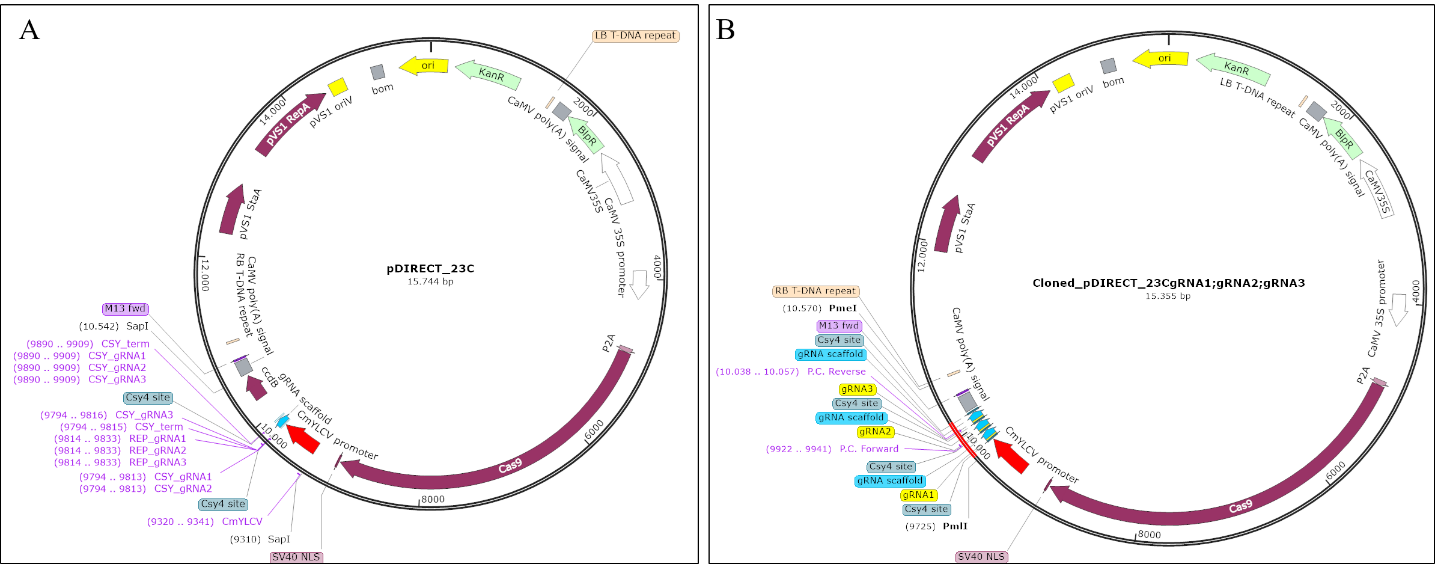


**Supplementary Figure 1.** pDIRECT_23C (Addgene 91140) plasmid and cloning oligonucleotides (CSY, REP, and CmYLCV) were given in A. PC primer’s binding sites and PmeI/PmlI restriction enzyme’s recognition sites have shown in cloned plasmid (B).


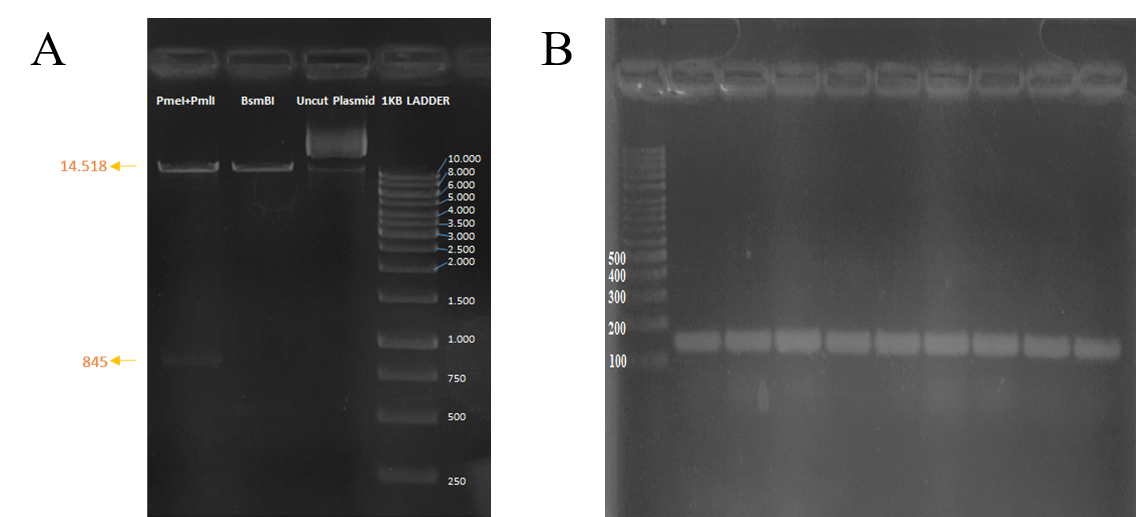


**Supplementary Figure 2.** Verification of the cloning plasmid with PmeI/PmlI (A) and PCR (B) with PC primers (135 bp)


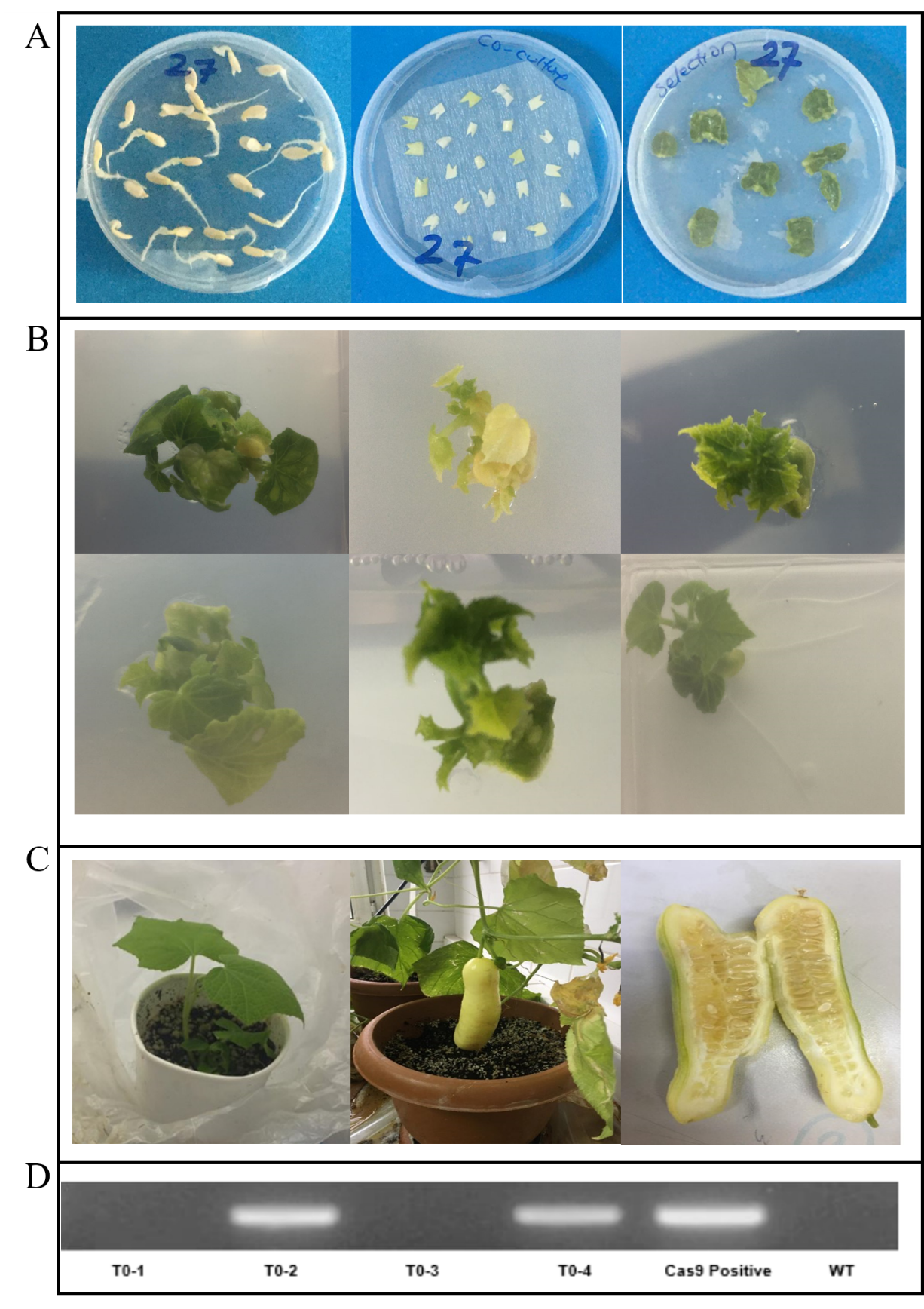


**Supplementary Figure 3.** Cotyledon explants from germinated ADR27 seeds and co-cultivation with EHA105 were given in A. Shot formation from transformed explants seems on selective MS-media, when chlorosis were detected on non-transformant plants, the roots were developed in transformed explants(B). T1 seeds were harvested from the regenerated plants after the acclimatization (C) and T-DNA confirmation with PCR by using Cas9 primers (D).


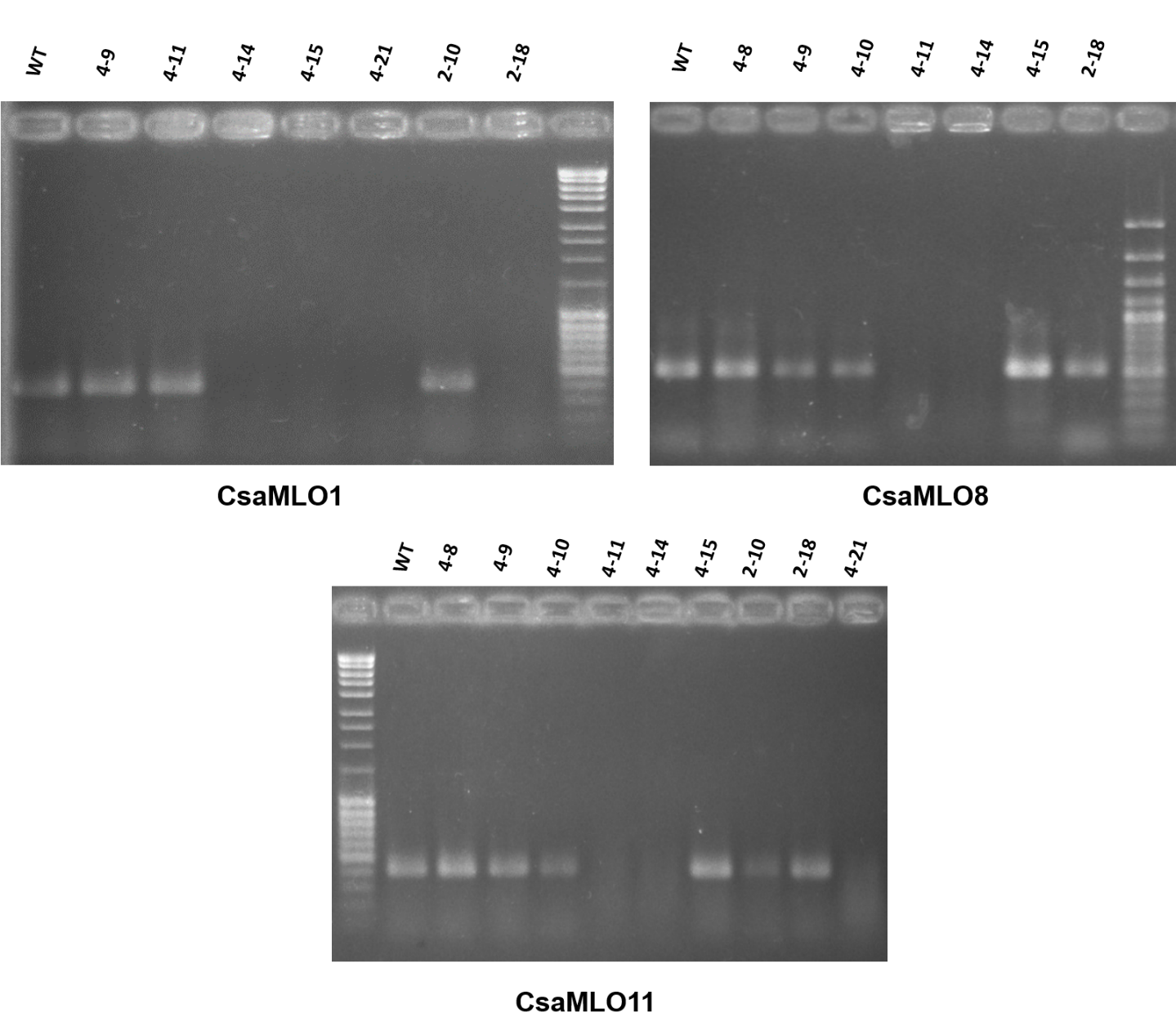


**Supplementary Figure 4.** Full-size gel image for carried out ACT-PCR to detect homozygous CsaMLO mutants

**Supplementary Table 1.**

Primers with their oligonucleotides that were used in the present study.

| **Oligonucleotide Name** | **Sequence** |
| --- | --- |
| **CSY_gRNA1** | TCGTCTCCCAAACTCCGCCGCTGCCTATACGGCAGTGAAC |
| **CSY_gRNA2** | TCGTCTCCTGCTCTGTTCCACTGCCTATACGGCAGTGAAC |
| **CSY_gRNA3** | TCGTCTCCGTCTCTTCCAAGCTGCCTATACGGCAGTGAAC |
| **REP_gRNA1** | TCGTCTCATTTGGCCGCTGCGTTTTAGAGCTAGAAATAGC |
| **REP_gRNA2** | TCGTCTCAAGCAGCGTACTTGTTTTAGAGCTAGAAATAGC |
| **REP_gRNA3** | TCGTCTCAAGACGCCGACATGTTTTAGAGCTAGAAATAGC |
| **CmYLCV** | TGCTCTTCGCGCTGGCAGACATACTGTCCCAC |
| **CSY_term** | TGCTCTTCTGACCTGCCTATACGGCAGTGAAC |
| **ACT-MLO1 F** | GGCCGCTGCGGGAGGAGAG |
| **ACT-MLO1R** | TCAGCACAGCCCTCCAAATTC |
| **ACT-MLO8 F** | CAGAGCAGCGTACTTTGGAAG |
| **ACT-MLO8R** | ACTACGGTGACTAGATGCGCC |
| **ACT-MLO11 F** | AAGAGACGCCGACATGGG |
| **ACT-MLO11R** | GAGGACTACATGGATGCCACG |
| **MLO1-SeqF** | GGGCAAGGACCAATAACGGA |
| **MLO1-SeqR** | TGGTGTGGACATCAGCACAG |
| **MLO8-SeqF** | ATTGGTTGCAGACCTTCTTA |
| **MLO8-SeqR** | CAGCCACTGCACAGATTT |
| **MLO11-SeqF** | CCCTACGCGTTCTAAAGTCA |
| **MLO11-SeqR** | TTAGGCTTTGTCCCACCGTC |
| **Cas9-F** | CGGAAACACCGATAGGCACT |
| **Cas9-R** | GATCATGTGAGCGAGAGCGA |
| **P.C. Forward** | TGGAACAGAGCAGCGTACTT |
| **R.C. Reverse** | ATGTCGGCGTCTCTTCCAAG |

**Supplementary Table 2.** Mutation rates in T1 generation

| Gene |  |  | No. of plants examined |  | No. of plants with mutations |  | Mutation rate (%) |  | Mutation Type |
| --- | --- | --- | --- | --- | --- | --- | --- | --- | --- |
| *CsaMLO1* |  |  | 96 |  | 24 |  | 25.00 |  | Deletion and Substitution |
| *CsaMLO8* |  |  | 96 |  | 2 |  | 2.08 |  | Deletion |
| *CsaMLO11* |  |  | 96 |  | 19 |  | 19.79 |  | Deletion |
